# Impacts of Corporate Social Responsibility on Employees’ Mental Fatigue: Employees’ Ethical Perspective

**DOI:** 10.3389/fpsyg.2022.918106

**Published:** 2022-06-15

**Authors:** Linlin Zheng, Wenzhuo Li, Amsalu K. Addis, Di Ye, Yashi Dong

**Affiliations:** ^1^Business School, Huaqiao University, Quanzhou, China; ^2^Business School, HoHai University, Nanjing, China; ^3^School of Business, Hubei University, Wuhan, China; ^4^Research Center of Open Economy, Hubei University, Wuhan, China

**Keywords:** corporate social responsibility, ethical egoism, mental fatigue, altruistic choice, corporate hypocrisy

## Abstract

With the rise of cost of living and coronavirus disease 2019 (COVID-19) global pandemic therewithal, finding reliable measures to reduce employees’ mental fatigue has become a great challenge. In this context, scholars have mainly focused on solutions for relieving employees’ mental fatigue from the perspective of human resource management but barely from employees’ ethical perspectives and that of internal and external corporate social responsibility (CSR) and employees’ ethics. This study uses hierarchical regression analysis and attempts to formulate and analyze the relationship between CSR, perceptions of corporate hypocrisy, and employees’ mental fatigue along with the mediating role of ethical egoism and altruistic choice. It also conceptualizes models and develops various hypotheses and theoretical logic. A total of 250 questionnaires were distributed, and 176 valid responses were subsequently gathered. The results show that employees’ mental fatigue significantly reduces when either internal or external CSR has a positive impact on employees’ altruistic choice and significantly increases either internal or external CSR has a negative effect on ethical egoism. Similarly, reducing perceptions of corporate hypocrisy can enhance the positive impact of external CSR on altruistic choice, which consequently reduces employees’ mental fatigue.

## Introduction

Because of the current coronavirus disease 2019 (COVID-19) global pandemic, medical staffs across the world have been treating confirmed cases, which appear to be increasing at an uncontrollable rate. Medical staffs have also been experiencing psychological breakdowns due to inadequate medical protection, consequently making the situation increasingly difficult. In 2020, the European Society of Medical Oncology released the results of a questionnaire survey that demonstrated that the mental fatigue suffered by medical staffs was severe, with 38% of the respondents having experienced severe mental fatigue and 66% of the respondents believing that they could not go back to the same working condition as before (Banerjee et al., 2021). Indeed, an increase of employees’ mental fatigue in the medical industry greatly lowers medical efficiency and ability to intervene in public health incidents. Therefore, organizations need to assume certain responsibilities to handle such matters, besides, corporate social responsibility (CSR) should also be required to strengthen employees commitment and engagement at work (Boutmaghzoute and Moustaghfir, 2021); this needs extensive attention for two simple reasons. On the one hand, employees bear heavy responsibility in their daily work (Collier and Esteban, 2007), and their attitudes and responses to CSR in turn encourage corporates to actively undertake social responsibility to keep employees motivated. On the other hand, employees are the most valuable resources of the corporates, and the corporate competition clearly depends on the job satisfaction, psychological strength, motivation, and well-being of employees. If a corporation provide extensive attention to CSR without corporate hypocrisy, it can obtain a competitive advantage (Karaibrahimoglu, 2010; Bahman et al., 2014).

Additionally, a good reputation for corporations that engage in CSR can help attract potential employees, keep them once hired, maintain their confidence and performance, and safeguard them from “mental fatigue” (Branco and Rodrigues, 2006). The disclosure of employees’ dissatisfaction with a corporation’s CSR would largely influence the development of the corporation’s potential resources and efficiency (Branco and Rodrigues, 2006). As a result, eliminating corporate hypocrisy, transparency, acknowledging social responsibility, and proper utilization of CSR decision-making concepts to deal with their employees’ mental fatigue could build a favorable image among employees. On the contrary, a poor reputation of corporations that engage in CSR activities result in a negative external stakeholder’s reaction, which could also have a direct or indirect influence on employees’ satisfaction with the corporation for various reasons including workload (Branco and Rodrigues, 2006). Employees observe the corporation to have a good reputation and flawless CSR decision-making. Similarly, corporations require employees to show allegiance and obedient behavior. Although employees have a considerable stake in the success of the corporation due to employment position and source of income, their dissatisfaction with either internal or external CSR will result in their quitting work. Thus, studying the relationship between CSR and employees’ mental fatigue from the ethical perspective of employees has very important theoretical and practical significance.

Furthermore, there is a phenomenon observed among some corporations where they pay more attention to work quality and efficiency based on the need of cost control. This causes employees to work harder, which leads to severe mental fatigue that in turn affects the operating conditions of corporations; thus, mental fatigue has become a concern for corporations. Research on employees’ mental fatigue began with the work of Freudenberger, who was the first to examine the concept from the perspective of clinical psychology (Freudenberger, 1975); according to this concept, employees develop negative mental states and behaviors because they exceed the limits of their work ability and cannot meet the goals and requirements set by a corporation. In addition, scholars described mental fatigue as experiencing psychological symptoms such as emotional exhaustion and depersonalization, resulting in low levels of accomplishment in individuals (Maslach et al., 2001). Similarly, emotional disorders and psychological burdens have a negative effect on work motivation and efficiency. Moreover, scholars articulated that employees’ mental fatigue directly affects the productivity and achievement of organizational goals (Wu and Wu, 2019). Therefore, this issue has attracted extensive attention from the academic community.

Early research on the causes of “mental fatigue” focused on the following two paradigms: first – exploring the causes at the individual level: age, gender, marital status, and educational background; second – investigating the causes at the situation level, such as working environment, professional characteristics, and leadership style. Since 1990s, research on the causes of employees’ mental fatigue has gained attention from the relationship perspective of employees and corporations’ micro-social responsibility, which reflects the company culture and working environment. For instance, Iverson et al. (1998) proposed that colleagues and managers’ support would mediate the relationship between role stress and mental fatigue (Iverson et al., 1998). Similarly, they found that support from colleagues lowers emotional exhaustion, while encouragement from leaders reduces depersonalization; both types of support allow employees to experience lower levels of mental fatigue and enhance personal accomplishment (Iverson et al., 1998). In addition, Maslach et al. (2001) identified a correlation between work stress and mental fatigue and observed that support from leaders is more important than support from colleagues in reducing employees’ mental fatigue. Besides internal organizational relationships that can influence employees’ mental fatigue, an increasing sense of CSR can impact employees’ mental fatigue to a certain extent (Shao et al., 2017). However, it is undeniable that several corporates are guilty of making profit at the expense of their employees (Glavas, 2016). To alleviate their operating pressure under environmental changes, these corporates frequently ignore their social responsibility and ethics and fail to utilize CSR decision-making concepts to deal with their employees’ mental fatigue as well as addressing the expectations of stakeholders and shareholders. A previous study has shown that individual ethical cognitive differences directly affect employees’ perspectives, prospects, and perceptions of organizational behavior, including the performance of CSR, and then affect employees’ work attitudes and behavioral performance (Ellis, 2009). Therefore, from the perspective of employees’ ethics, this study can more intuitively investigate and demonstrate the overall relationships between CSR and employees’ mental fatigue in the workplace.

Although corporations have concerns about employees’ welfare and demands, their main intention is to encourage employees to work harder and prioritize the corporation’s efficiency goals (Richter et al., 2020), which puts employees under pressure and makes them exhausted in fulfilling the corporation’s goals. Some scholars have noted that a corporation’s responsibility toward its stakeholders’ interests and environmental protection positively affects its employees’ sense of organizational identity, enhances employees’ self-worth and self-esteem, encourages employees to adopt positive attitudes and behaviors, and reduces their mental fatigue (Farooq et al., 2016). Besides, employees have different perceptions based on CSR orientation when facing internal and external stakeholder issues (Farooq et al., 2016). This is specifically reflected in the finding by Scheidler et al. (2019): a corporation’s excessive preference for external CSR has a negative impact on employees, while its preference for internal CSR has a positive effect on employees. The imbalance between internal and external CSR leads to different emotional responses among employees. It thus be a practical and effective way to study the antecedents of employees’ mental fatigue from a CSR perspective. To date, research on the relationship between different aspects of CSR and employees’ mental fatigue as well as the impact of CSR on employees’ well-being has remained minimal.

This study argues that employees’ personal ethics and perceptions of corporate hypocrisy are the keys to resolving the issues of employees’ mental fatigue as well as promoting the corporation’s development factors. Personal ethical ideologies can be classified into altruistic choices with the motivation to improve the welfare of others and ethical egoism with the motivation to promote personal well-being (Forsyth, 1992). Furthermore, corporations’ external pursuit for community harmony and global well-being promotes consumers’ altruistic choices, while their internal pursuit for product uniqueness and quality excellence fosters consumers’ egoism. The ethical egoism in question shows that CSR affects different aspects of customers’ ethical ideologies. When employees perceive that a corporation actively assumes CSR, they will have a stronger dedication, tend to make altruistic choices, and thus engage in less counterproductive behavior. Similarly, Kung et al. (2021) studied employees’ daily emotional exhaustion, health, positive mood, and other pro-social behaviors and concluded that employees’ health was positively associated with their positive mood and negatively related to their emotional exhaustion; they further examined the relationship between organizational behavior and employees’ ethical ideologies (Kung et al., 2021). However, relevant studies are insufficient, and there is no clear answer as to how corporations can guide employees to make different ethical choices through CSR decision-making tools to improve their behavior and reduce mental fatigue.

Regarding the study of employee behavior, scholars often conduct additional research on employees’ perceptions of corporate hypocrisy. Several studies from the literature marked that when a corporation’s values are inconsistent with its actual operation, employees perceive that as corporate hypocrisy (Argyris, 1974; Runkel et al., 1976). Moreover, when a corporate actively promotes external CSR but neglects internal CSR, or shows inconsistency between its statements and performances, employees develop an aversion toward the corporate; this in turn leads to negative emotions and counter-productive behavior (Yang and Diefendorff, 2010).

Most studies have focused on the prevention of employees’ mental fatigue, yet they lack a more comprehensive understanding concerning how and why employees’ mental fatigue sets in and the impacts of both internal and external CSR on employees’ well-being. Thus, many companies cannot find a correct way to fulfill or compensate both internal and external CSR and their overall impact on employees. This study contributes to filling that gap by exploring the relationship between internal and external CSR and employees’ mental fatigue and attempts to answer the following fundamental research questions through a series of studies and methodological approaches:

1. What is the relationship between internal and external CSR and the ethical ideology of employees?
2. Does internal or external CSR affect employees’ mental fatigue by influencing their ethical ideology?
3. How do employees’ perceptions of corporate hypocrisy affect their ethical ideology?

Furthermore, this study connects CSR, employees’ ethical ideology, perceptions of corporate hypocrisy, and employees’ mental fatigue; creatively integrates cross-professional knowledge, constructs innovative models, and new theoretical logic; and lays the foundation for future scholars on related subjects.

This study uses stakeholder theory and important sociological theories to jointly analyze organizational factors and employee behavior in the workplace. The two theories lay a solid theoretical and practical foundation for the formation of research logic and frameworks, hypothesis proposals, and research development. Under the stakeholder theory, employees are important stakeholders in the company. As participants in the company’s operation, employees’ behavior, attitude, and cognition can be influenced by organizational factors. Simultaneously, employees also influence the operation and development of the company; understanding this vicious circle of process provides a sufficient theoretical and practical basis for researchers in the field. Under sociological theories, this study mentioned four important theories, which are logically interconnected and complement each other with regard to explanatory variables, in order to provide theoretical and practical support and understand the interactions and relationships among employees, the corporation, and the environment.

## Theoretical Foundation and Hypotheses Development

### Stakeholder Theory

As a management concept, CSR has always been inseparable from stakeholders (Tokoro, 2007; Žukauskas et al., 2018). CSR can generally be understood as the path through which a company performs a balance of social, environmental, and economic imperatives while upholding the anticipation of stakeholders (O’Riordan and Fairbrass, 2008). In 1963, the Stanford Research Institute put forward the stakeholder theory whereby an interest group that supports the survival of a corporation divides it into stakeholders and non-stakeholders based on their impact on the survival of the corporation (Berman et al., 1999; O’Riordan and Fairbrass, 2008). Since then, the definition of stakeholders has led to much controversy (Tokoro, 2007; Žukauskas et al., 2018). Freeman’s stakeholder theory states that a corporation’s stakeholders include those that can be affected by the corporation and further classified stakeholders into internal and external stakeholders, which in turn, has different levels such as primary and secondary (Freeman, 1984). Frost defined direct and indirect stakeholders according to the market transaction relationship between stakeholders and corporations (Frost, 1995), whereas Clarkson (1994, 1995) described primary and secondary stakeholders based on the closeness of the relationship between a group and a corporation. In addition, Sheldon suggested in his book *The Philosophy of Management* that a corporation’s business objectives should consider not only the interests of stakeholders but also those of other shareholders (Sheldon, 2003). Compared with previous research, the current stakeholder classification affords greater flexibility, yet it is not possible to find a standard and unified clarification as well as classification. Overall, the method of dividing stakeholders by the boundary of a corporation has gradually become a mainstream subject of discussion recognized by the academic community (Farooq et al., 2016). Freeman and Dmytriyev (2017) proved the correlation between CSR research and stakeholder theory: they both have the same goal of value creation, and they also found that some disputes of CSR can be overcome by balancing stakeholders’ demands. Moreover, an increasing number of scholars have adopted this method of dividing stakeholders based on the classification of social responsibility (Miles, 2017). The application of stakeholder theory on CSR can help scholars establish a coherent and comprehensive theoretical basis in the field of social problems in management (Dmytriyev et al., 2021). Nonetheless, corporations should ensure good stakeholder management from both the inside and outside of the corporation, which prompted this study to divide CSR into internal and external based on the classification of stakeholders. As obvious, employees are fundamental internal stakeholders and have a direct stake in the corporation.

### Sociological Theories

The social exchange theory is also known as the theory of reciprocity (Cropanzano and Mitchell, 2005). According to the theory, once employees feel that a company cares about them, they will further contribute to the corporation, become proactive in their work, seek personal performance improvement, and concurrently bolster the company to achieve its organizational goals. Scholars have used social exchange theory to analyze the behavioral motivation of employees participating in CSR activities in the corporations. For example, Slack et al. (2015) pointed out that the social exchange mechanism encourages employees to return a favor to the corporations when they receive benefits from CSR activities. Social exchange theory is the basis for the conceptualization and operation of internal CSR. It has a strong connection with internal CSR between corporation and employees (Mory et al., 2016), which partially explains the relationship between internal CSR and employees’ mental fatigue.

Social comparison theory is one among the sociological theories and is alternatively called the fairness theory (Jiang, 2016). Employees’ enthusiasm toward their work is closely related to their perception of the fairness of a corporation’s distribution policy. Employees compare their treatment with the treatment of external stakeholders and use the results of the comparison to evaluate the value of their behavior. If there is a large deviation from the self-set psychological value, the deviation will be reduced by employees’ behavioral change. When employees find out that the treatment they receive is better than that of external stakeholders, the employees believe that they enjoy a higher degree of recognition and respect. In return, employees may increase their efforts and extend their working time; this simultaneously reduces mental fatigue at the workplace. Lemke and Vries (2021) pointed out that social comparison theory can tap into the potential positive or negative effects on individual behavior to explain the reasons for behavior change. This theory provides ideas and a basis for this study to deeply explore the hypothesis related to the causes of employees’ mental fatigue.

Additionally, social learning theory states that human behavior is the result of the interaction between human cognition and the environment and that human behavior will gradually become spontaneous by humans learning and imitating from role models. CSR could imperceptibly guide employees’ altruistic choices and encourage them to actively make greater efforts toward fulfilling organizational goals. In recent years, numerous scholars have used this theory to study individual antisocial and pro-social behavior, employees’ workplace deviant behavior, and interpret CSR into personal actions (Kabiri et al., 2020; Qi et al., 2020; Tekleab et al., 2020). Social learning theory provides a comprehensive social framework to explain individual motivation for this study.

Furthermore, social cognitive theory posits that the environment, cognitive factors, and human behavior constitute a continuous, dynamic, interactive, and reciprocal relationship. There is a two-way relationship between these factors, i.e., individual behavior is a combination of cognition and environmental factors (Wu and Wu, 2019). According to the social cognitive theory, the evaluation of employees’ internal and external images of their company is highly related to their behavior choices and value creation (Almeida and Coelho, 2019). When an individual’s perception clashes with existing ideas, they will experience cognitive dissonance (Dragone et al., 2019). Consequently, employees reduce the pressure caused by dissonance through behavioral change to maintain balanced perceptions. This theory provides theoretical support for explaining the possibility of corporate social responsibility and employee performance.

These four theories complement each other with regard to explanatory variables. Social learning theory emphasizes the influence of the environment on individual behavior, while social cognitive theory emphasizes the influence of individual cognition in the environment on their behavior. Thus, the two theories effectively illustrate the process of the influence of organizational factors on employees’ behavior. Besides, social exchange theory and social comparison theory are the most influential conceptual paradigms in organizational behavior, which complement the employees’ behavioral adjustment process and the factors affecting their work performance. Social exchange theory clarifies the reciprocal exchange process between a corporation and its employees, and it explains the impact of the actual economic resource development of the company and the rewards provided by the company to employees based on their attitude and behavior at work as well as their perception of organizational behavior. Social comparison theory is the concept that employees determine their own social and personal worth by evaluating their own abilities, attitudes, and traits in the corporation in comparison to others and then adjusting their behavior accordingly. These two theories also complement each other, which makes the hypothesis derivation logic of this study more rigorous and coherent.

### Hypothetical Reasoning

#### Relationship Between CSR and Employees’ Ethical Ideology and Mental Fatigue

According to the social exchange theory and social comparison theory, if a corporation provides good salary and welfare benefits, creates a good organizational atmosphere, and broadens promotion channels, then its employees will in return make altruistic choices in favor of the corporation, such as by increasing their working time and maintaining the company’s image (Akarsu et al., 2020). This demonstrates the presence of social exchange relationship between internal CSR and individual behavior. Corporations and employees form a mutually beneficial relationship, which should allow incentives to guide employees making altruistic ethical decisions. Based on this, the following hypothesis is proposed:

*H1*: When internal CSR prompts employees to make altruistic choices, employees’ mental fatigue will reduce.

According to social learning theory, it cannot be ruled out that corporations attach great importance to internal CSR and establish an organizational culture considering the greater internal interests (Kim et al., 2020); this encourages employees to increase personal interests and form a benignly competitive relationship with their colleagues and external stakeholders. A continuous competitive relationship makes employees excessively concerned about the gain and loss of their interests and fails to secure the expected interest would affect employees’ enthusiasm for work, which increases the possibility of them developing mental fatigue. Based on this point, the following hypothesis is proposed:

*H2*: When internal CSR guides employees to promote ethical egoism, employees’ mental fatigue will increase.

According to social cognitive theory, the active undertaking of external CSR helps establish a responsible corporation, promotes a strong integration of a corporation’s values and employees’ personal values, and creates an altruistic organizational structure that encourages employees to take the initiative of assuming more work responsibility (Saleem et al., 2021). Moreover, the higher an corporation’s degree of fulfillment to external CSR, the more helpful it would be in improving employees’ work performance, learning initiatives, and altruistic behavior (Littlejohn and Foss, 2009). In other words, employees gain a sense of honor and belong to a collective family in the context of a corporation, motivating them to make more altruistic choices and their workplace engagement (Dutton et al., 1994). This will imperceptibly instigate employees’ altruistic choice, which encourages them to make greater efforts toward fulfilling organizational goals and reduces mental fatigue. Based on this, the following hypothesis is proposed:

*H3*: When external CSR promotes employees to make altruistic choices, employees’ mental fatigue will reduce.

Employees compare their remuneration with that of stakeholders outside the corporation to judge whether the corporation’s recognition of their behavior value is reasonable (Jiang, 2016). When there is a large deviation between expectation and actual perception, employees will take actions to reduce the deviation and balance the psychological gap. However, in this case, they only have the leeway of deciding to lower their job involvement, resulting in an increasing mental fatigue. Based on this, the following hypothesis is proposed:

*H4*: When external CSR enhances employees’ ethical egoism, employees’ mental fatigue will increase.

#### The Role of Perceptions of Corporate Hypocrisy

Corporate hypocrisy occurs when a company promise to do something in the name of corporate social responsibility but then does something quite opposite (Miao and Zhou, 2020; Chang and Cho, 2021; Tiwari et al., 2021). When a corporation prioritizes internal CSR than external CSR, employees perceive it as the corporation being more willing to bring direct benefits or allocate resources to the corporation as well as its employees (Wu et al., 2020a). Contrarily, when a corporation operates for the purpose of making a profit, employees imitate the hypocritical behavior of corporation, which could affect their feedback psychology and voluntary contributions (Kim et al., 2020) and result in egoism (Dragone et al., 2019). Besides, when employees realize that there is a dissonance between their own remuneration and the corporate’s external interests, they assume that the corporation ignores their contribution, which leads to staff turnover, egoism, and rebellion (Miller and Flores, 2007). Conversely, the greater the employees trust in the company, the more they would be committed to and focused on promoting company interests (Thau et al., 2007). In particular, perceptions of corporate hypocrisy weaken employees’ altruistic choices and tend to enhance their ethical egoism. Based on this fact, the following hypothesis is proposed:

*H5*: Stronger perceptions of corporate hypocrisy weaken the positive impact of internal CSR on employees’ altruistic choices and enhance the positive impact of internal CSR on employees’ ethical egoism.

According to social comparison theory, when a corporation pays too much attention to external social responsibility while ignoring internal social responsibility, employees will think that the corporation attaches a low value to their work, and they will thus perceive the corporation as hypocritical (Miller et al., 2015). With stronger perceptions of corporate hypocrisy, employees’ sense of identity is reduced, and they would therefore pay more attention to their own interests (ethical egoism) within the company (Sidgwick, 2011). It means that external CSR does not subtly stimulate employees’ altruistic choices (Thyer and Myers, 1998). Based on the above analysis, the following hypothesis is advanced:

*H6*: Stronger perceptions of corporate hypocrisy weaken the positive impact of external CSR on employees’ altruistic choices and enhance the positive impact of external CSR on employees’ ethical egoism.

#### Theoretical Model

**Figure 1:**
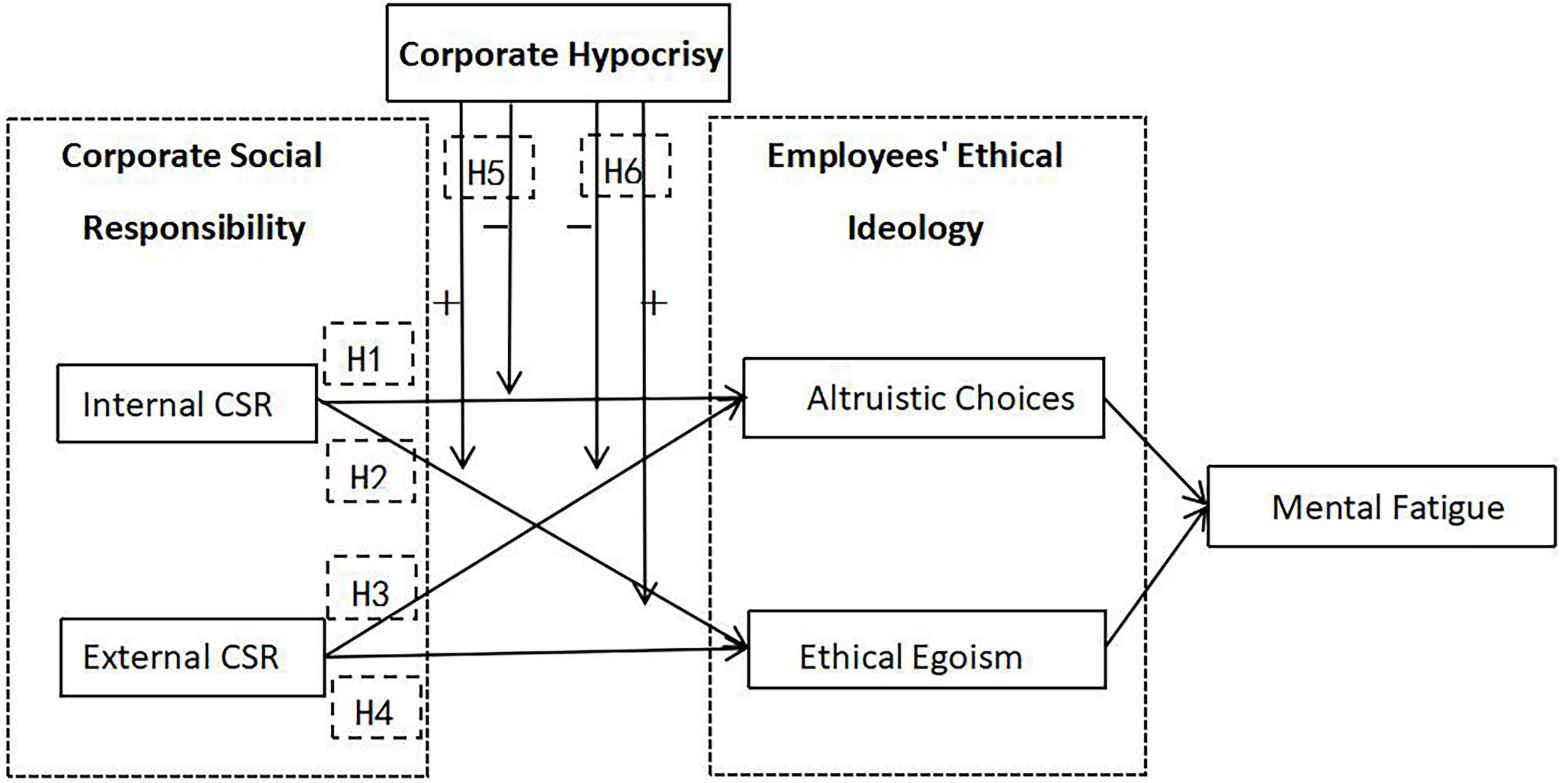
Theoretical model.

It is assumed that the internal and/or external CSR affects employees’ mental fatigue by influencing either employees’ altruistic choice or ethical egoism (Jia et al., 2019). Corporate hypocrisy, as a moderating variable, changes the direction and degree of the relationship between CSR and employee’s ethical ideologies. The research model is shown in Figure 1.

## Data and Methods

### Research Design and Data Collection

This study included responses of employees from different industries and corporations who are working in various employment positions in Chinese provinces, namely Henan, Gansu, Shanxi, and Fujian province. Data were gathered from randomly selected employees by adopting qualitative semi-structural interviews and questionnaires in two phases. The first stage of interview with managers laid the foundation for the design of questionnaires, whereas the second stage of interview with employees is to confirm whether the feedback of the questionnaire can be replicated in the small-scale questionnaire survey. The first stage is a small-scale pre-survey. A total of 250 questionnaires were distributed, and 176 valid responses were collected. The questionnaire was divided into six parts. The first part is to mainly understand the basic information of the respondents, and the other five parts focus on the information about employees’ mental fatigue, internal CSR, external CSR, perceptions of corporate hypocrisy, ethical egoism, and altruistic choice (discussed in the section below). Based on the collected data, this study analyzed and clarified the relationship among the employees’ mental fatigue, internal CSR, external CSR, perceptions of corporate hypocrisy, ethical egoism, and altruistic choice. The study utilized the SPSS software to conduct the statistical analysis of the data and to scientifically analyze the mechanism of internal CSR and external CSR on employees’ mental fatigue from a theoretical and empirical perspective. Furthermore, available literature has also been reviewed.

The responses were measured using a 5-point Likert-type scale, with the corresponding score for responses to each question survey ranging as “strongly agree” (5 points), “agree” (4 points), “uncertain” (3 points), “disagree” (2 points), and “strongly disagree” (1 point). As an ethical consideration, the respondents volunteered to participate in the study and provided written consent before answering the questionnaire. They were told that they could discontinue their participation at any time without any consequences. To ensure anonymity, personal information was kept in a master file that was separate from the dataset used for the study analysis.

We chose respondents from various levels of gender, age, working years, and working position. Regarding the respondents’ gender composition, there were more women than men by a slight margin. There were no deviations in the gender characteristics. The respondents were grouped by age, namely, 24 years and below (18.18%), 25–34 years (23.86%), 35–44 years (33.52%), and 45–54 years (22.73%). The age distributions of the surveyed employees were uniform and covered all age groups, which reduces the data bias caused by a concentration in younger age groups. For the respondents’ working years, while more than half has worked for more than 9 years, participants in the other age groups were evenly distributed, and the experienced employees have a clear understanding of the implementation of internal and external CSR, which improved the accuracy of data collection. Similarly, the proportions of respondents in different working positions were random. Conclusively, the questionnaire’s data collection could be deemed reliable and comprehensive.

### Data Measurement

#### Scale of Internal CSR (Four Items)

Scale of internal CSR refers to the measurement of Fortune Index in Griffin and Mahon’s research (Griffin and Mahon, 1997), selecting the indicators of “talent attraction, cultivation and use” to measure the internal employee responsibility performance. We integrate scales and make final measurement indicators that include information transparency and attractive remuneration incentives for employees, the working environment and management of employees, promotion and salary adjustment, and training.

#### Scale of External CSR (Five Items)

Scale of external CSR refers to the reactive defensive accommodative and proactive (RDAP) model developed by Clarkson (1995) and fortune index. According to the fortune index, the evaluation of corporate community and environmental social responsibility is determined, and the indicators such as innovation, product and service quality, and community and environmental responsibility are selected to evaluate the performance of corporates’ responsibility and important external stakeholders such as industry, customers, community and society; referring to the RDAP model, selecting indicators such as environmental protection, social donation, education investment, community, and competitive relationship to supplement the assessment of corporates’ responsibility for public stakeholders and market competition participation. After comprehensive consideration, the final measurement indicators include corporate charity and education aid, products, publicity information and after-sales services, fair competition with competitors, employment opportunities and facilities for the community, and environmental protection and technological innovation for the country.

#### Scale of Perception of Corporate Hypocrisy (Three Items)

Scale of perception of corporate hypocrisy refers to scales developed by Wagner et al. (2009) and Fassin and Buelens (2011). Wagner et al.’s (2009) scale focuses on the individual’s perception of inconsistent information, such as inconsistent CSR behavior and ability and inconsistent matching between behavior and events. While Fassin and Buelens (2011) focus on the corporate’s own attributes, such as CSR behavior motivation, execution strength, and communication degree. We integrate scales and make final measurement indicators include employees’ attitude toward CSR, employees’ awareness of the purpose of CSR, and employees’ understanding of the motivation for CSR.

#### Scale of Altruistic Choices (Four Items)

According to the subscale of altruistic behavior in the Chinese organizational citizenship behavior scale, which was developed by Farh et al. (1997) and MacKenzie et al. (1991), final measurement indicators include employees’ willingness to share information with colleagues, willingness to help new colleagues, work engagement and voluntary sacrifice, and the balance between their values and interest.

#### Scale of Ethical Egoism (Four Items)

According to the employee self-interest behavior scale, adapted by Gkorezis and Bellou (2016), and the dimension of “personal initiative” in the organizational citizenship behavior scale, developed by MacKenzie et al. (1991), final measurement indicators include knowledge retention, pursuing personal development, giving priority to personal interests, and unwillingness to cooperate in a team.

#### Scale of Mental Fatigue (Five Items)

This part refers to the Maslach Burnout Inventory (MBI) and improved version, proved by Dolan et al. (2015), final measurement indicators include employees’ work enthusiasm, recognition for the employees’ work value and significance, the possibility of employees achieving their career goals, the attitude of employees toward challenges at work, and willingness of employees to overcommit their time to a task.

### Data Analysis

As illustrated in Table 1, external corporate social responsibility is significantly positively correlated with altruistic choice (*p* < 0.01) and negatively correlated with mental fatigue (*p* < 0.01). Similarly, mental fatigue was negatively correlated with altruistic choice (*p* < 0.01) and positively correlated with egoistic choice (*p* < 0.01). The correlation analysis lays a foundation for further research about the causal relationship between variables.

**Table 1:** Means, standard deviations and correlation coefficient analyses of the variables.

	M	SD	1	2	3	4	5	6	7	8	9	10
1. Gender	1.63	0.486	1									
2. Age	2.66	1.073	0.016	1								
3. Working years	3.70	1.605	0.038	0.683^***^	1							
4. Working position	1.75	1.240	−0.062	0.09	0.258^***^	1						
5. Internal CSR	2.100	0.836	−0.107	−0.006	−0.215^***^	−0.155^**^	1					
6. External CSR	2.078	0.746	−0.12	−0.081	−0.231^***^	−0.102	0.649^***^	1				
7. Enterprise hypocrisy	3.210	1.124	−0.019	0.068	0.104	−0.041	−0.218^***^	−0.117	1			
8. Altruistic choice	2.156	0.792	−0.129^*^	−0.031	−0.177^**^	−0.209^***^	0.570^***^	0.497^***^	−0.123	1		
9. Ethical egoism	3.459	0.967	−0.027	0.102	0.111	−0.005	−0.205^***^	−0.108	0.445^***^	−0.346^***^	1	
10. Mental fatigue	3.430	1.025	0.05	0.031	0.139^*^	0.133^*^	−0.314^***^	−0.207^***^	0.612^***^	−0.304^***^	0.400^***^	1

^*^p < 0.01, ^**^p < 0.05, ^***^p < 0.1.

#### Reliability Analyses and Validity Test

The validity and reliability of the scales used in research are important factors that enable the research to yield healthy results (Roberts and Priest, 2006; Sürücü and Maslakci, 2020). The primary purpose is to test the accuracy of the study model and to demonstrate the rigor and trustworthiness of qualitative and quantitative research. In this case, this study used Cronbach’s *α* and confirmatory factor analysis (CFA) for reliability analyses and validity test, respectively. Cronbach’s *α* was developed by Lee Cronbach in 1951 to provide a measure of the internal consistency of a test or scale; it ranges between 0 and 1, with higher values indicating that the survey or questionnaire is more reliable (Tavakol and Dennick, 2011). It is a way to measure the internal consistency and reliability of a questionnaire or survey by comparing the amount of shared variance or covariance among the items (Taber, 2018). SPSS 25.0 was used to test the reliability of the collected data. Thus, Cronbach’s *α* coefficient for internal CSR, external CSR, perceptions of corporate hypocrisy, altruistic choice, ethical egoism, and mental fatigue were 0.961, 0.953, 0.958, 0.930, 0.923, and 0.952, respectively (see Table 2).

**Table 2:** Reliability statistics for each variable of the research model.

Items	Questionnaire contents	Factor loading	AVE	Cronbach’s *α* coefficient
Internal CSR	Internal information disclosure and investor incentives	0.818	0.669	0.961
	Work environment optimization	0.799		
	Promotion and salary	0.850		
	Stage training	0.822		
External CSR	Corporate charity and public welfare assistance	0.850	0.748	0.953
	Products, publicity, and after-sales service	0.893		
	Play fairly with competitors	0.872		
	Community employment opportunities and facility construction	0.829		
	Environmental protection and technological innovation	0.879		
Corporate hypocrisy perception	Attitude toward corporate social responsibility	0.836	0.730	0.958
	The cognition of the purpose of corporate social responsibility	0.858		
	Understanding of the motivation for corporate social responsibility	0.869		
Altruistic choice	The willingness to share the information	0.814	0.687	0.930
	The will to help new colleagues	0.864		
	Work participation and voluntary sacrifice	0.791		
	A balance between values and interests	0.846		
Ethical egoism	Knowledge retention	0.865	0.742	0.923
	The pursuit of personal development	0.872		
	Personal interests are preferred	0.842		
	Do not want to team together	0.867		
Mental fatigue	Low enthusiasm for work	0.884	0.745	0.952
	Low recognition of work value and significance	0.883		
	The probability of achieving your career goals is low	0.904		
	Passive attitude toward the challenges and difficulties at work	0.835		
	Low willingness to commit to a task effectively and efficiently	0.806		
	Low enthusiasm for work	0.884		

The result clearly demonstrated that the Cronbach’s α value for each dimension are greater than the acceptable reliability, *α* = 0.80 (Taber, 2018), which confirmed the reliability or consistency of the study data.

Confirmatory factor analysis (CFA), on the contrary, is a type of factor analysis used whether the designed scale (questionnaire) is valid for data collection and to test a factor regarding unmeasured sources of variability (Hoyle, 2012). The CFA is a method for calculating structural validity and the measurement part of the models (Prudon, 2015). Therefore, by using the CFA, covariance matrixes were computed and fit indices were carried out, then the general structure of the research questionnaire content and study model validity were checked. In Table 3, the results of the CFA of the research variables were obtained by using the data science analysis platform SPSSAU.

**Table 3:** Confirmatory factor analysis.

Common indicators	*χ* ^2^	*df*	GFI	RMR	CFI	NFI	NNFI	RMSEA
Value	536.094	260	0.806	0.042	0.945	0.900	0.937	0.078
Other indicators	TLI	IFI	–	–	–	–	–	–
Value	0.937	0.946	–	–	–	–	–	–

GFI, goodness of fit; RMR, root mean square residual; CFI, comparative fit index; NFI/NNFI, (non) normed fit index; RMSEA, root mean squared error of approximation; TLI, Tucker Lewis index; IFI, incremental fit index.

The standard of fit indices in the structural equation modeling (SEM): GFI should be >0.95 (Schermelleh-Engel et al., 2003), and the study result shows 0.806, just close to the acceptable score, this is because the GFI is known to depend on the sample size (Mulaik et al., 1989). RMR should be <0.08; represents the square-root of the difference between the residuals of the sample covariance matrix and the hypothesized model (Hooper et al., 2008). CFI should be >0.90 (Fan et al., 1999). NFI/NNFI/TLI should be >0.90 (Byrne, 1994; Schumacker and Lomax, 1996), and an NFI/NNFI of 0.95 indicates the model of interest improves the fit by 95 (Hooper et al., 2008). RMSEA should be <0.08 and a value closer to 0 represent a good fit (Chen et al., 2008). IFI > 0.90 is a good fit, but the index can exceed 1 (Hooper et al., 2008). The result in the CFA table clearly demonstrated that all the mentioned values are within the standard range. Overall, the research questionnaire content and study model is well constructed.

#### Common Method Deviation Test

The Harman single factor test was used to determine whether there is a common method deviation in the collected data. All items on the six-variable measurement scale were included in the exploratory factor analysis. The results showed that there were six factors with a characteristic root greater than one, and the maximum factor variance interpretation rate was 39.723% (see Table 4). It indicates that there is no serious common method deviation in this study.

**Table 4:** Total variance analysis.

Component	Initial eigenvalues			Extraction sums of squared loadings		
	Total	% of variance	Cumulative%	Total	% of variance	Cumulative%
1	9.931	39.723	39.723	9.931	39.723	39.723
2	5.096	20.383	60.106	5.096	20.383	60.106
3	2.46	9.839	69.945	2.46	9.839	69.945
4	1.734	6.938	76.882	1.734	6.938	76.882
5	1.322	5.286	82.168	1.322	5.286	82.168
6	1.044	4.177	86.345	1.044	4.177	86.345
...						
25	0.056	0.225	100			

If the total variance extracted by one factor exceeds 50%, there is common method bias in the study. Therefore, these data indicate that there is no problem with common method bias since the total variance extracted by one factor is 39.723%, and it is less than the recommended threshold of 50%.

## Results

### The Mediating Effect of Ethical Ideology of Employees

As demonstrated in Table 5, the results of the regression analysis for internal CSR → altruistic choice showed that internal CSR has a positive impact on altruistic choice (*β* = 0.508, *p* < 0.05). On the contrary, the results of the regression analysis of internal CSR → mental fatigue showed that internal CSR has a significant negative impact on mental fatigue (*β* = −0.347, *p* < 0.01).When internal CSR → mental fatigue (*β* = −0.347, *p* < 0.01) compared with the results of the regression analysis of internal CSR → mental fatigue after altruistic choice was added as intermediary (*β* = −0.235, *p* < 0.05), although both have a significant negative impact on employees’ mental fatigue, after adding altruistic choice as an intermediate variable, internal CSR has a reduced impact on mental fatigue. This asserted that there was a mediating effect between internal CSR and mental fatigue. The above result generally demonstrates that H1 is consistent.

**Table 5:** Mediation effects of employee ethical selection.

Variable	Mental fatigue							Altruistic choice			Ethical egoism		
	M 0–1	M 1–1	M 1–2	M 2–1	M 2–2	M 3–1	M 4–1	M 0–2	M 1–3	M 4–2	M0-3	M 3–2	M 4–3
Controlled variable													
Gender	0.108	0.046	0.018	0.067	0.025	0.088	0.103	−0.218^*^	−0.128	−0.133	−0.066	−0.111	−0.089
Age	−0.098	−0.031	−0.030	−0.076	−0.058	−0.064	−0.097	0.102	0.005	0.056	0.039	0.087	0.051
Working years	0.116	0.052	0.048	0.082	0.070	0.048	0.067	−0.110^**^	−0.016	−0.040	0.055	0.009	0.037
Work position	0.082	0.061	0.043	0.076	0.045	0.077	0.088	−0.110^**^	−0.079^*^	−0.098^**^	−0.027	−0.042	−0.030
Independent variable													
Internal CSR		−0.347^***^	−0.235^**^			−0.252^***^			0.508^***^			−0.250^***^	
External CSR				−0.234^**^	−0.080		−0.182^*^			0.487^***^			−0.128
Mediating variable													
Altruistic choice			−0.220^**^		−0.316^***^								
Ethical egoism						0.379^***^	0.409^***^						
F	1.678	4.189	4.155	2.345	3.387	8.42	7.475	4.143	18.074	13.371	0.678	2.076	0.857
R^2^	0.038	0.11^***^	0.129^***^	0.065^**^	0.107^***^	0.230^***^	0.21	0.08^***^	0.347^***^	0.282^***^	0.016	0.058^*^	0.025

^*^p < 0.1, ^**^p < 0.05, ^***^p < 0.01.

The results of the regression analysis of internal CSR → ethical egoism showed that internal CSR has a negative impact on ethical egoism (*β* = −0.250, *p* < 0.01). However, the result of the regression analysis of internal CSR → mental fatigue after ethical egoism was added as an intermediate variable, and internal CSR was articulately demonstrated to have an increased statistically significant negative impact on mental fatigue (*β* = −0.252, *p* < 0.01). Concurrently, employees’ tendency toward ethical egoism increases mental fatigue. The result also clarifies that ethical egoism has a partial mediating effect between internal CSR and mental fatigue. Therefore, the result indicates that H2 is logical.

The results of the regression analysis of external CSR → altruistic choice showed that external CSR has a positive impact on altruistic choice (*β* = 0.487, *p* < 0.01). On the contrary, altruistic choice → mental fatigue has an increased significant negative impact (*β* = −0.316, *p* < 0.01). When these results were compared with the regression analysis results of external CSR → mental fatigue (*β* = −0.234, *p* < 0.05) after altruistic choice was added as an intermediate variable, the result clearly indicates that external CSR has a reduced impact on mental fatigue. Therefore, we can understand that the intermediate variable (altruistic choice) has a complete mediating effect between external CSR and mental fatigue. As a result, H3 is compatible.

The result of regression analysis of external CSR → ethical egoism (*β* = −0.128, *p* < 0.1), it indicated that external CSR has a negative impact on ethical egoism. When this result compared with the result of regression analysis of external CSR → mental fatigue (*β* = *−*0.234, *p* < 0.05), it indicated that external CSR has a reduced significant impact on ethical egoism but increased impact on mental fatigue. Besides, ethical egoism has a significant positive impact on mental fatigue (*β* = 0.409, *p* < 0.01), and there is no mediating effect of ethical egoism existed between CSR and mental fatigue. Therefore, H4 is proved to be inconsistent.

### Moderating Effect of Perceptions of Corporate Hypocrisy

From the perspective of internal CSR, by taking altruistic choice as the outcome variable and adding internal CSR, perceptions of corporate hypocrisy, and the interaction between them, it can be observed that the main effect was not affected by the addition of the interaction item. However, after all three variables were added, the analysis results showed that the coefficient of perceptions of corporate hypocrisy and its interaction was insignificant. This signifies that there was no moderating effect of perceptions of corporate hypocrisy between internal CSR and altruistic choice. When taking ethical egoism as the outcome variable, it was found that “internal CSR* perception of hypocrisy” had no significant effect on ethical egoism, which signifies that perceptions of corporate hypocrisy were not associated with a significant adjustment between internal CSR and ethical egoism. Consequently, H5 is shown to be inconsistent (Table 6).

**Table 6:** Moderating effects of perception of hypocrisy.

	Altruistic choice							Ethical egoism						
	M 0–4	M 4–1	M 4–2	M 4–3	M 5–1	M 5–2	M 5–3	M 0–5	M 6–1	M6-2	M 6–3	M 7–1	M 7–2	M 7–3
Gender	−0.218^**^	−0.128	−0.128	−0.132	−0.133	−0.138	−0.158	−0.066	−0.111	−0.068^*^	−0.059	−0.089	−0.054	−0.034
Age	0.102	0.005	0.005	0.004	0.056	0.056	0.027	0.039	0.087	0.073	0.077	0.051	0.053^*^	0.082
Working years	−0.110^**^	−0.016	−0.016	−0.015	−0.04	−0.036	−0.031	0.055	0.009	−0.006	0.007	0.037	0.009	−0.004
Work position	−0.110^**^	−0.079^*^	−0.079^*^	−0.081^*^	−0.098^**^	−0.102^**^	−0.113^***^	−0.027	−0.042^*^	−0.011	−0.007	−0.03	−0.003	−0.009
Internal CSR		0.508^***^	0.506^***^	0.502^***^					−0.258^**^	−0.142^**^	−0.13			
External CSR					0.487^***^	0.479^***^	0.381^***^					−0.128	−0.068	−0.028
Perception of hypocrisy			−0.007	−0.015		−0.053	−0.105^**^			0.355^***^	0.375^***^		0.372^***^	0.423^***^
Internal CSR *Perception of hypocrisy				−0.018							0.046			
External CSR *Perception of hypocrisy							−0.168^***^							0.166^**^
F	4.143	18.074	14.979	12.79	13.371	11.382	11.391	0.678	2.076	7.817	6.747	0.857	7.348	7.137
R^2^	0.088^***^	0.347^***^	0.347^***^	0.348^***^	0.282^***^	0.288^***^	0.322^***^	0.016	0.058^*^	0.217^***^	0.219^***^	0.025	0.207^***^	0.229^***^

^*^p < 0.1, ^**^p < 0.05, ^***^p < 0.01.

On the contrary, from the perspective of external CSR, by taking altruistic choice as the outcome variable, external CSR, perceptions of corporate hypocrisy, and their interaction item were included in the regression. The moderating variables of perceptions of corporate hypocrisy and the interaction item were significant. There was a back-moderating effect on the relationship between external CSR and altruistic choice. Additionally, when taking ethical egoism as the outcome variable, both hypocrisy perception and “external CSR* perception of hypocrisy” have significant positive effects on ethical egoism. This signifies that perceptions of corporate hypocrisy played a moderating role in the relationship between external CSR and ethical egoism, and perceptions of corporate hypocrisy moderates the relationship between external CSR and ethical egoism. Therefore, H6 is proven to be logical and sound.

### Simple Slope Test of Moderating Effect

**Figure 2:**
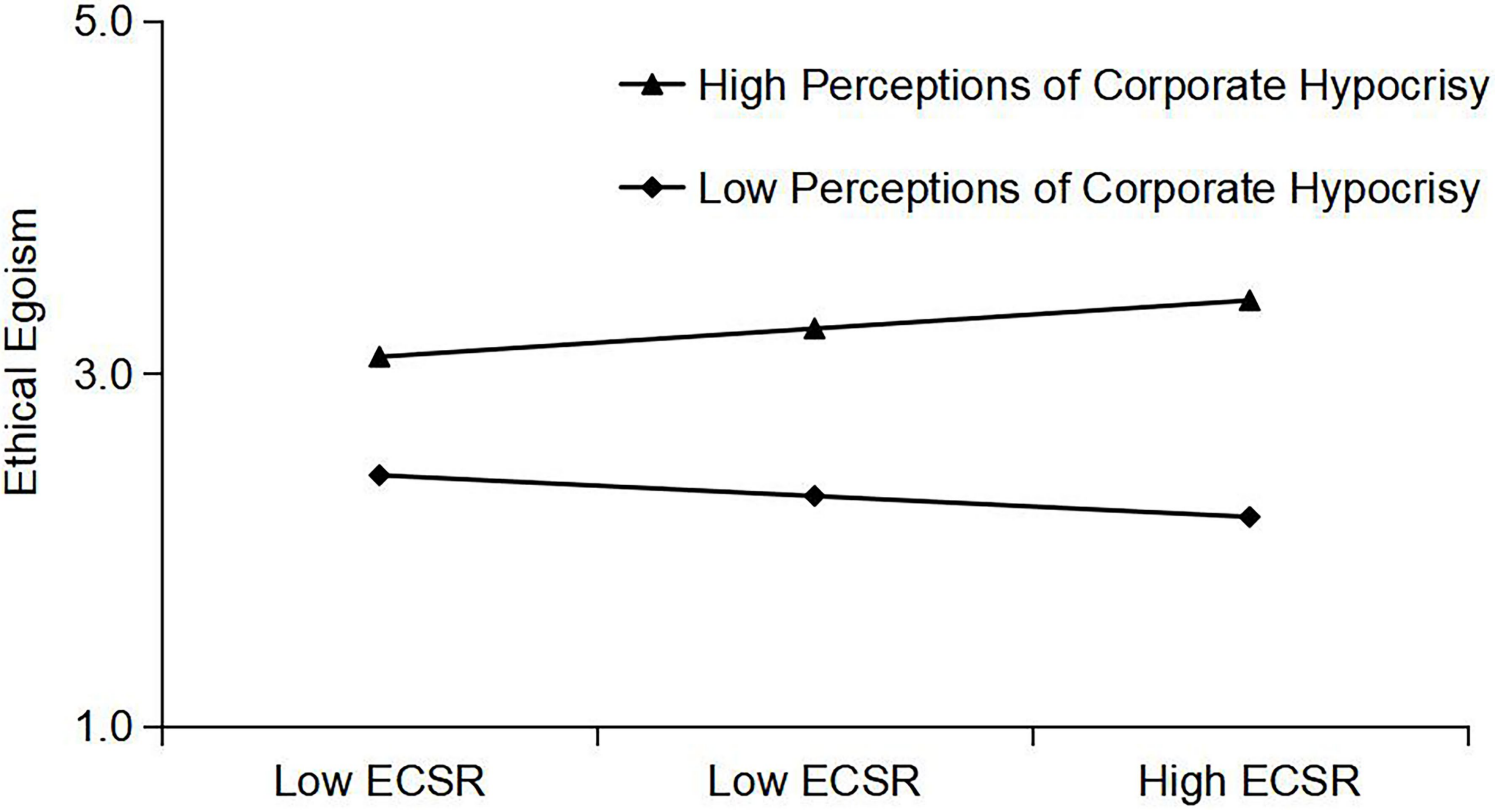
External CSR and ethical egoism: moderating effect of perceptions of corporate hypocrisy.

Figure 2 shows that perceptions of corporate hypocrisy positively moderated the relationship between external CSR and employees’ ethical egoism; high perceptions of corporate hypocrisy gave rise to a high tendency of ethical egoism in employees, which further proves that H6 is logical.

**Figure 3:**
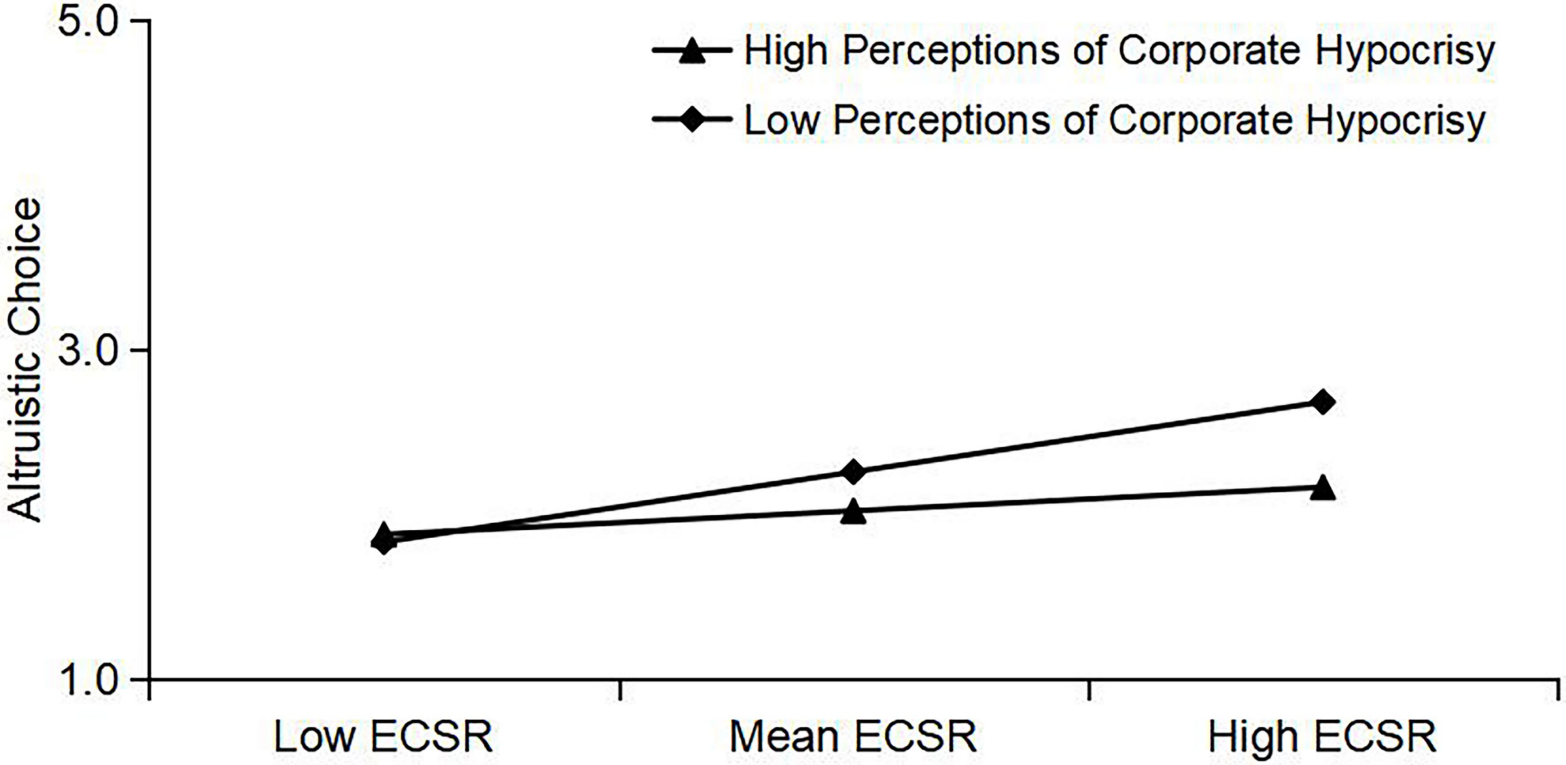
External CSR and altruistic choice: moderating effect of perceptions of corporate hypocrisy.

Incidentally, Figure 3 illustrates that perceptions of corporate hypocrisy negatively moderate the relationship between external CSR and employees’ altruistic choices. In other words, high perceptions of corporate hypocrisy influence employees to have a low tendency of making altruistic choices, which demonstrates that H6 is consistent. This study complements the theory that external CSR may affect employees’ mental fatigue due to the influence of factors such as employees’ personal morality and ethical pursuits.

**Figure 4:**
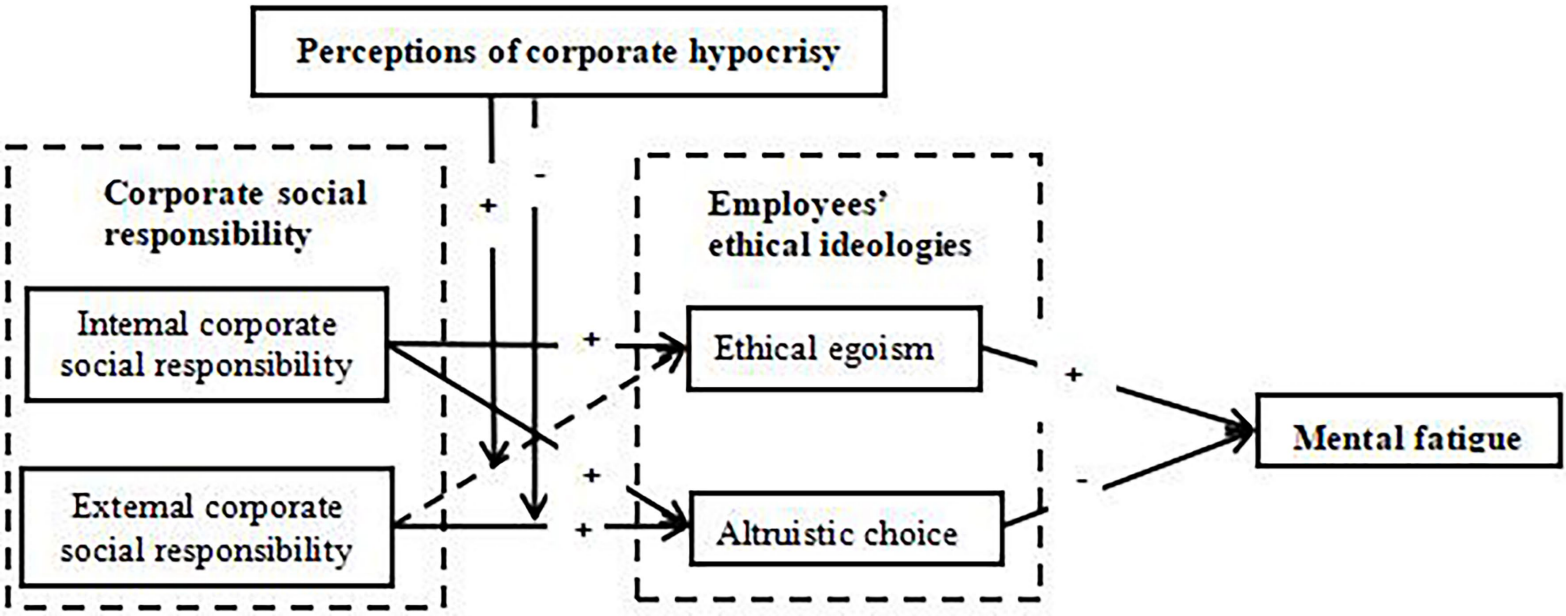
Conceptualization and validation of the study model.

Corporate hypocrisy does not moderate the relationship between internal social responsibility and ethical egoism, as well as the relationship between internal social responsibility and altruistic choice. Based on the above results, the research model of this study can be conceptualized as given in Figure 4.

## Discussion

### Theoretical Implications

Many studies regarding the impact of CSR on employees have mostly focused on the attitude of employees’ toward organizational identity and emotional commitment. While some scholars have also paid attention to the impact of organizational factors on employees’ mental fatigue, few have studied the impact of CSR on employees’ mental fatigue without directly analyzing CSR and mental fatigue in a conceptual model (Thompson et al., 2019; Wu et al., 2020b). However, this study focuses on the relationship between CSR and mental fatigue, analyzes the integration of CSR and employees’ mental fatigue, introduces employees’ ethical ideologies as an intermediary variable, connects the concepts of CSR and employees’ mental fatigue from different aspects, and explores how internal and external CSR affect ethical ideology.

Additionally, this study uses perceptions of corporate hypocrisy as a moderating variable, which makes the research path more accurate, and provides a reference for future research on CSR and related fields. It also analyzes the influences of CSR on employees’ mental fatigue from the perspective of employees’ ethics, aiming to enrich the research on the relationship between CSR and employee behavior, which is of great significance to theoretical and practical research innovation and development. Theoretically, this research contributed to the understanding of the multiple interactive relationships between CSR (internal and external) and mental fatigue: internal CSR → altruistic choices → mental fatigue, internal CSR → ethical egoism → mental fatigue, external CSR → altruistic choices → mental fatigue, external CSR → ethical egoism → mental fatigue, and further expanded the literature on the consequence of CSR and the antecedents of mental fatigue. Furthermore, this study found that perceptions of corporate hypocrisy strengthened the relationship between CSR and ethical egoism. Moreover, it introduces corporate hypocrisy factors that intensify the influence path of CSR on employee ethical ideology, which elevates the cross-field research on the topic related to employees’ mental fatigue, and creatively puts forward a new path of the impact of internal and external social responsibility on employees’ mental fatigue.

### Managerial Implications

From a managerial perspective, this study offers various insights for corporations to reduce employees’ mental fatigue, such as closely associating with human resource management development to improve working environments. In addition, it provides a concept for corporate managers to develop their employees’ proclivity for making altruistic choices in order to reduce mental fatigue and strengthen ethical ideologies. In this regard, ethical ideologies are considered significant in the achievement of companies’ CSR practices. Moreover, managers should also strengthen the internal and external CSR framework in order to convince their employees, reduce perceptions of corporate hypocrisy, and safeguard employees’ mental health.

First, managers in corporations can improve their employees’ working conditions and mental fatigue by implementing sound human resource management (HRM) practices. In the practice of HRM, for example, managers can direct employees to voluntarily participate in social services, as well as advise and motivate other employees to make positive ethical choices as assessment indicators of job accomplishment and advancement. Similarly, this study urges corporations to reduce employees’ negative perceptions related to corporate hypocrisy by increasing transparency in information disclosure, and reducing the information gap between employees and corporations. For instance, using a social responsibility column on a corporation’s official website, documents and information related to the performance of the CSR can be shared and updated, and internal communications can be attended to using the intranet or newspapers, thus improving the performance of internal CSR, increasing employee satisfaction, establishing complaint mechanisms, and reducing mental fatigue caused by poor information can be achieved. This activity axiomatically serves to ameliorate employees’ perceptions of corporate hypocrisy, lessen the gap between employees’ personal income and salary, enhance the sense of organizational identity (Miao and Zhou, 2020), improve the working atmosphere (Almeida and Coelho, 2019), and reduce psychological fatigue (Iverson et al., 1998).

Second, this study also encourages corporations to proportionally implement internal and external CSR so as to reduce employees’ mental fatigue. Corporations should increase the influence of internal and external CSR on employees’ altruistic choices. When driven by a harmonious and friendly working environment and the unified goal of maximizing organizational interests, employees engage in altruistic choices more frequently than ethical egoism. It should be noted that CSR should not pay too much attention to external CSR or ignore internal CSR, because a corporation’s internal CSR is closely related to individual interests and development and directly affects the ethical ideologies of employees. Corporations should pay attention to the rights and requirements of employees, provide them with appropriate treatment, create safe working environments, and even guide them in career development, which will minimize their mental fatigue, improve the overall performance of the corporation, and secure long-term sustainable development.

Meanwhile, it is crucial to stress that in the implementation of CSR, corporations should consider balancing the needs of diverse beneficiary groups under internal and external social responsibility, rather than focusing solely on commercial profits. Corporations should earnestly consider the true demands of their internal and external stakeholders when carrying out their tasks. For example, although employees assist corporations in making product supply more efficient, high-quality outcomes, low-carbon emission, and so on, corporations should also see employees as key resources and consider providing corresponding incentives. Moreover, when managers in a corporation develop an internal and external social responsibility framework, it is important to aim at connecting the objectives of product improvement, business model innovation, and environmental sustainability with the needs of internal and external stakeholders.

In conclusion, the findings recommend that corporations cultivate an ethical atmosphere, pay attention to employees’ ethical ideology, enhance the consistency of the company’s values and goals, and reduce the influences, which can lead to mental fatigue. A reasonable ethical atmosphere within a corporation is an advantageous way to reduce employees’ mental fatigue as it can help employees understand common values and organizational goals, which can weaken perceptions of corporate hypocrisy, guide and improve employees to make more altruistic choices, and allow altruistic choices to become part of employees’ spontaneous ethical consciousness. Moreover, corporations should make their organizational ethical standards clear so the corporation can be quickly recognized and accepted by employees, which will be reflected in employees’ altruistic behavior and may effectively enhance employees’ pursuit of personal contributions at work.

### Limitations and Future Research

This study fills the literature gaps in related fields and helps relevant organizations and corporations understand the impact of internal and external CSR on employees’ behavior, specifically mental fatigue. However, this study has several limitations. The research did not focus on a specific industry or corporation, so the results may not represent the situation in all industries. Moreover, the study did not examine whether the impact of CSR on employees’ mental fatigue was different in different cultures. In other words, the scope of the sample collection was limited; cross-border and cross-cultural comparative studies could be carried out to increase the universality and applicability of the conclusions. Therefore, the results may not be applicable to groups with different cultural backgrounds. Incidentally, future research could be based on quantitative research combined with a fuzzy set qualitative comparative analysis method (Villalba-Ríos et al., 2022) to enhance the universality and persuasiveness of the research conclusion.

## Conclusion

In the growing field of mental health research, this study attempted to formulate and analyze the relationship between CSR and mental fatigue, along with the mediating role of ethical ideologies, as well as the moderating role of perceptions of corporate hypocrisy. The study found that internal CSR has a significant positive impact on mental fatigue when ethical egoism is evoked and a significant negative impact on mental fatigue when altruistic choice is evoked. Additionally, altruistic choice has a significant mediating effect between CSR and mental fatigue, and ethical egoism has a partial mediating effect between CSR and mental fatigue. Moreover, perceptions of corporate hypocrisy enhance the positive guiding effect of CSR on employees’ ethical egoism, while employees with egoism are more prone to mental fatigue. This study suggests that corporations should focus on employees’ ethical ideologies by balancing different dimensions of CSR with appropriate ways to make altruistic choices become a part of employees’ spontaneous ethical consciousness. Thus, employees’ mental health can be safeguarded while achieving and sustaining organizational goals, this is a strategy that could be beneficial to all parties involved.

## Data Availability Statement

The original contributions presented in the study are included in the article/supplementary material, further inquiries can be directed to the corresponding author.

## Author Contributions

LZ: conceptualization, methodology, software, and writing–reviewing and editing. AA: writing–reviewing and editing and supervision. DY: investigation, data curation, and validation. WL and YD: writing–original draft preparation. All authors contributed to the article and approved the submitted version.

## Funding

The author received national and university financial support for the research as follows: National Social Science Fund Project “Research on Co-benefit Business Model Innovation Based on Enterprise’ Economic Benefit and Social Responsibility” (20CGL003).

## Conflict of Interest

The authors declare that the research was conducted in the absence of any commercial or financial relationships that could be construed as a potential conflict of interest.

## Publisher’s Note

All claims expressed in this article are solely those of the authors and do not necessarily represent those of their affiliated organizations, or those of the publisher, the editors and the reviewers. Any product that may be evaluated in this article, or claim that may be made by its manufacturer, is not guaranteed or endorsed by the publisher.

## References

[ref1] Akarsu T. N. Foroudi P. Melewar T. (2020). What makes Airbnb likeable? Exploring the nexus between service attractiveness, country image, perceived authenticity and experience from a social exchange theory perspective within an emerging economy context. Int. J. Hosp. Manag. 91:102635. doi: 10.1016/j.ijhm.2020.102635

[ref2] Almeida M. d. G. M. C. Coelho A. F. M. (2019). The antecedents of corporate reputation and image and their impacts on employee commitment and performance: the moderating role of CSR. Corp. Reputation Rev. 22, 10–25. doi: 10.1057/s41299-018-0053-8

[ref3] Argyris C. (1974). Theory in practice: increasing profession. Soc. Serv. Rev. 49, 292–293. doi: 10.1086/643248

[ref4] Bahman S. P. Kamran N. Mostafa E. (2014). Corporate social responsibility: a literature review. Afr. J. Bus. Manag. 8, 228–234. doi: 10.5897/AJBM12.106

[ref5] Banerjee S. Lim K. H. J. Murali K. Kamposioras K. Punie K. Oing C.. (2021). The impact of COVID-19 on oncology professionals: results of the ESMO resilience task force survey collaboration. *ESMO Open* 6:100058. doi: 10.1016/j.esmoop.2021.100058, PMID: 33601295 PMC7900705

[ref6] Berman S. L. Wicks A. C. Kotha S. Jones T. M. (1999). Does stakeholder orientation matter? The relationship between stakeholder management models and firm financial performance. Acad. Manag. J. 42, 488–506. doi: 10.5465/256972

[ref7] Boutmaghzoute H. Moustaghfir K. (2021). Exploring the relationship between corporate social responsibility actions and employee retention: a human resource management perspective. Hum. Sys. Manag. 40, 789–801. doi: 10.3233/HSM-211202

[ref8] Branco M. C. Rodrigues L. L. (2006). Corporate social responsibility and resource-based perspectives. J. Bus. Ethics 69, 111–132. doi: 10.1007/s10551-006-9071-z

[ref9] Byrne B. M. (1994). Structural Equation Modeling with EQS and EQS/Windows. Thousand Oaks, CA: SAGE Publications.

[ref10] Chang H. E. Cho S. H. (2021). The influence of social support on the relationship between emotional demands and health of hospital nurses: a cross-sectional study. Healthcare 9:115. doi: 10.3390/healthcare9020115, PMID: 33498995 PMC7912004

[ref11] Chen F. Curran P. J. Bollen K. A. Kirby J. Paxton P. (2008). An empirical evaluation of the use of fixed cutoff points in RMSEA test statistic in structural equation models. Socio. Meth. Res. 36, 462–494. doi: 10.1177/0049124108314720, PMID: 19756246 PMC2743032

[ref12] Clarkson M. B. (1994). A Risk Based Model of Stakeholder Theory. Toronto: The Centre for Corporate Social Performance and Ethics

[ref13] Clarkson M. B. (1995). A stakeholder framework for analyzing and evaluating corporate social performance. Acad. Manag. Rev. 20, 92–117. doi: 10.2307/258888

[ref14] Collier J. Esteban R. (2007). Corporate social responsibility and organizational commitment. Bus. Ethics: A Eur. Rev. 16, 19–33. doi: 10.1111/j.1467-8608.2006.00466.x

[ref15] Cropanzano R. Mitchell M. S. (2005). Social exchange theory: an interdisciplinary review. J. Manag. 31, 874–900. doi: 10.1177/0149206305279602

[ref16] Dmytriyev S. D. Freeman R. E. Hrisch J. (2021). The relationship between stakeholder theory and corporate social responsibility: differences, similarities, and implications for social issues in management. J. Manag. Stud. 58, 1441–1470. doi: 10.1111/joms.12684

[ref17] Dolan E. D. Mohr D. Lempa M. Joos S. Fihn S. D. Nelson K. M.. (2015). Using a single item to measure burnout in primary care staff: a psychometric evaluation. J. Gen. Intern. Med. 30, 582–587. doi: 10.1007/s11606-014-3112-6, PMID: 25451989 PMC4395610

[ref18] Dragone M. Esposito C. De Angelis G. Affuso G. Bacchini D. (2019). Pathways linking exposure to community violence, self-serving cognitive distortions and school bullying perpetration: a three-wave study. Int. J. Environ. Res. Public Health 17:188. doi: 10.3390/ijerph17010188, PMID: 31888112 PMC6981769

[ref19] Dutton J. E. Dukerich J. M. Harquail C. V. (1994). Organizational images and member identification. Adm. Sci. Q. 39, 239–263. doi: 10.2307/2393235

[ref20] Ellis A. D. (2009). The impact of corporate social responsibility on employee attitudes and behaviors. Acad. Manag. Ann. Meeting Proc. 2009, 1–6. doi: 10.5465/AMBPP.2009.44251836

[ref21] Fan X. Wang L. Thompson B. (1999). Effects of sample size, estimation methods, and model specification on structural equation modeling fit indexes. Struc. Eq. Mod. 6, 56–83. doi: 10.1080/10705519909540119

[ref22] Farh J. L. Earley P. C. Lin S. C. (1997). Impetus for action: a cultural analysis of justice and organizational citizenship behavior in Chinese society. Adm. Sci. Q. 42, 421–444. doi: 10.2307/2393733

[ref23] Farooq O. Rupp D. E. Farooq M. (2016). The multiple pathways through which internal and external corporate social responsibility influence organizational identification and multifoci outcomes: the moderating role of cultural and social orientations. Acad. Manag. J. 60, 954–985. doi: 10.5465/amj.2014.0849

[ref24] Fassin Y. Buelens M. (2011). The hypocrisy-sincerity continuum in corporate communication and decision making: a model of corporate social responsibility and business ethics practices. Manag. Decis. 49, 586–600. doi: 10.1108/00251741111126503

[ref25] Forsyth D. R. (1992). Judging the morality of business practices: the influence of personal moral philosophies. J. Bus. Ethics 11, 461–470. doi: 10.1007/BF00870557

[ref26] Freeman R. E. (1984). Strategic Management: A Stakeholder Approach. Boston: Pitman Publishing.

[ref27] Freeman R. E. Dmytriyev S. (2017). Corporate social responsibility and stakeholder theory: learning from each other. Symphonya. Emerg. Iss. Manag. 2, 7–15. doi: 10.4468/2017.1.02freeman.dmytriyev

[ref28] Freudenberger H. J. (1975). The staff burn-out syndrome in alternative institutions. Psychol. Psychother. 12, 73–82. doi: 10.1037/h0086411

[ref29] Frost F. A. (1995). The use of stakeholder analysis to understand ethical and moral issues in the primary resource sector. J. Bus. Ethics 14, 653–661. doi: 10.1007/BF00871346

[ref30] Gkorezis P. Bellou V. (2016). The relationship between workplace ostracism and information exchange: the mediating role of self-serving behavior. Manag. Decis. 54, 700–713. doi: 10.1108/MD-09-2015-0421

[ref31] Glavas A. (2016). Corporate social responsibility and employee engagement: enabling employees to employ more of their whole selves at work. Front. Psychol. 7:796. doi: 10.3389/fpsyg.2016.00796, PMID: 27303352 PMC4886691

[ref32] Griffin J. J. Mahon J. F. (1997). The corporate social performance and corporate financial performance debate: twenty-five years of incomparable research. Soc. Sci. Electro. Pub. 36, 5–31. doi: 10.1177/000765039703600102

[ref33] Hooper D. Coughlan J. Mullen M. R. (2008). Structural equation modelling: guidelines for determining model fit. Electron. J. Bus. Res. Methods 6, 53–60. doi: 10.21427/D7CF7R

[ref34] Hoyle R. H. (2012). “Confirmatory factor analysis,” in Encyclopedia of Social Science Research Methods. eds. Lewis-Beck M. Bryman A. Liao T. (Thousand Oaks, CA: SAGE Publications), 169–175.

[ref35] Iverson R. D. Olekalns M. Erwin P. J. (1998). Affectivity, organizational stressors, and absenteeism: a causal model of burnout and its consequences. J. Vocational Behav. 52, 1–23. doi: 10.1006/jvbe.1996.1556

[ref36] Jia Y. Yan J. Liu T. Huang J. (2019). How does internal and external CSR affect employees’ work engagement? Exploring multiple mediation mechanisms and boundary conditions. Int. J. Environ. Res. Public Health 16(14), 2476. doi: 10.3390/ijerph16142476, PMID: 31336754 PMC6678673

[ref37] Jiang W. (2016). Limited public resources allocation model based on social fairness using an extended VIKOR method. Kybernetes 45, 998–1012. doi: 10.1108/K-05-2014-0108

[ref38] Kabiri S. Shadmanfaat S. Smith H. Cochran J. (2020). A social learning model of antisocial coaching behavior. Int. J. Offender Therapy Comp. Criminol. 64, 860–879. doi: 10.1177/0306624X19899608, PMID: 31928277

[ref39] Karaibrahimoglu Y. Z. (2010). Corporate social responsibility in times of financial crisis. Afr. J. Bus. Manag. 4, 382–389.

[ref40] Kim H. Rhou Y. Topcuoglu E. Kim Y. G. (2020). Why hotel employees care about corporate social responsibility (CSR): using need satisfaction theory. Int. J. Hosp. Manag. 87:102505. doi: 10.1016/j.ijhm.2020.102505

[ref41] Kung Y. T. Chi S. C. Chen Y. C. Chang C. M. (2021). Using residual dynamic structural equation modeling to explore the relationships among employees’ self-reported health, daily positive mood, and daily emotional exhaustion. Healthcare 9:93. doi: 10.3390/healthcare9010093, PMID: 33477488 PMC7831058

[ref42] Lemke M. Vries R. (2021). Operationalizing behavior change theory as part of persuasive technology: a scoping review on social comparison. Front. Comp. Sci. 3:656873. doi: 10.3389/fcomp.2021.656873

[ref43] Littlejohn S. W. Foss K. A. (2009). “Organizational identity theory,” in Encyclopedia of Communication Theory. eds. Littlejohn S. W. Foss K. A. (Thousand Oaks, CA: SAGE Publications Incorporated), 717–718.

[ref44] MacKenzie S. B. Podsakoff P. M. Fetter R. (1991). Organizational citizenship behavior and objective productivity as determinants of managerial evaluations of salespersons’ performance. Organ. Behav. Hum. Decis. Process. 50, 123–150. doi: 10.1016/0749-5978(91)90037-T

[ref45] Maslach C. Schaufeli W. B. Leiter M. P. (2001). Job burnout. Annu. Rev. Psychol. 52, 397–422. doi: 10.1146/annurev.psych.52.1.397 11148311

[ref46] Miao Q. Zhou J. (2020). Corporate hypocrisy and counterproductive work behavior: a moderated mediation model of organizational identification and perceived importance of CSR. Sustainability 12, 1847. doi: 10.3390/su12051847

[ref47] Miles S. (2017). “Stakeholder theory classification, definitions and essential contestability,” in Stakeholder Management (Business and Society 360, Vol. 1). ed. Wasieleski D. M. (Bingley, UK: Emerald Publishing Limited), 21–47.

[ref48] Miller M. K. Flores D. (2007). “Social comparison theory,” in The Blackwell Encyclopedia of Sociology. ed. Ritzer G. (Chichester: John Wiley & Sons).

[ref49] Miller M. K. Reichert J. Flores D. (2015). “Social comparison,” in The Blackwell Encyclopedia of Sociology. ed. Ritzer G. (Chichester: John Wiley & Sons).

[ref50] Mory L. Wirtz B. W. Göttel V. (2016). Factors of internal corporate social responsibility and the effect on organizational commitment. Int. J. Hum. Resour. Manag. 27, 1393–1425. doi: 10.1080/09585192.2015.1072103

[ref51] Mulaik S. A. James L. R. Van Alstine J. Bennett N. Lind S. Stilwell C. D. (1989). Evaluation of goodness-of-fit indices for structural equation models. Psychol. Bull. 105, 430–445. doi: 10.1037/0033-2909.105.3.430

[ref52] O’Riordan L. Fairbrass J. (2008). Corporate social responsibility (CSR): models and theories in stakeholder dialogue. J. Bus. Ethics 83, 745–758. doi: 10.1007/s10551-008-9662-y

[ref53] Prudon P. (2015). Confirmatory factor analysis as a tool in research using questionnaires: a critique. Comp. Psychol. 4:03.CP.4.10. doi: 10.2466/03.CP.4.10

[ref54] Qi L. Liu B. Mao K. (2020). Spare the rod and spoil the child? A study on employee workplace deviant behavior. Nankai Bus. Rev. Int. 11, 1–22. doi: 10.1108/NBRI-03-2018-0019

[ref55] Richter U. H. Shirodkar V. Shete N. (2020). Firm-level indicators of instrumental and political CSR processes – a multiple case study. Eur. Manag. J. 39, 279–290. doi: 10.1016/j.emj.2020.07.004

[ref56] Roberts P. Priest H. (2006). Reliability and validity in research. Nurs. Stand. 20, 41–45. doi: 10.7748/ns.20.36.41.s58, PMID: 16872117

[ref57] Runkel P. J. Argyris C. Schon D. A. (1976). Theory in practice: increasing professional effectiveness. J. High. Educ. 47, 113. doi: 10.2307/1978718

[ref58] Saleem M. Qadeer F. Mahmood F. Han H. Giorgi G. Ariza-Montes A. (2021). Inculcation of green behavior in employees: a multilevel moderated mediation approach. Int. J. Environ. Res. Public Health 18:331. doi: 10.3390/ijerph18010331, PMID: 33466298 PMC7794897

[ref59] Scheidler S. Edinger-Schons L. M. Spanjol J. Wieseke J. (2019). Scrooge posing as Mother Teresa: how hypocritical social responsibility strategies hurt employees and firms. J. Bus. Ethics 157, 339–358. doi: 10.1007/s10551-018-3788-3

[ref60] Schermelleh-Engel K. Moosbrugger H. Müller H. (2003). Evaluating the fit of structural equation models: tests of significance and descriptive goodness-of-fit measures. Met. Psychol. Res. 8, 23–74.

[ref61] Schumacker R. E. Lomax R. G. (1996). A Beginner’s Guide to Structural Equation Modeling. 4th Edn. New York: Psychology Press.

[ref62] Shao B. Cardona P. Ng I. Trau R. (2017). Are prosocially motivated employees more committed to their organization? The roles of supervisors' prosocial motivation and perceived corporate social responsibility. Asia Pac. J. Manag. 34, 951–974. doi: 10.1007/s10490-017-9512-5

[ref63] Sheldon O. (2003). The Philosophy of Management. London: Routledge.

[ref64] Sidgwick H. (2011). The Methods of Ethics. Cambridge: Cambridge University Press. 7.

[ref65] Slack R. E. Corlett S. Morris R. (2015). Exploring employee engagement with (corporate) social responsibility: a social exchange perspective on organisational participation. J. Bus. Ethics 127, 537–548. doi: 10.1007/s10551-014-2057-3

[ref66] Sürücü L. Maslakci A. (2020). Validity and reliability in quantitative research. Bus. Manag. Stu. Int. J. 8, 2694–2726. doi: 10.15295/bmij.v8i3.1540

[ref67] Taber K. S. (2018). The use of Cronbach’s alpha when developing and reporting research instruments in science education. Res. Sci. Educ. 48, 1273–1296. doi: 10.1007/s11165-016-9602-2

[ref68] Tavakol M. Dennick R. (2011). Making sense of Cronbach’s alpha. Int. J. Med. Educ. 2, 53–55. doi: 10.5116/ijme.4dfb.8dfd, PMID: 28029643 PMC4205511

[ref69] Tekleab A. G. Reagan P. M. Do B. Levi A. Lichtman C. (2020). Translating corporate social responsibility into action: a social learning perspective. J. Bus. Ethics 171, 741–756. doi: 10.1007/s10551-020-04447-y

[ref70] Thau S. Crossley C. Bennett R. J. Sczesny S. (2007). The relationship between trust, attachment, and antisocial work behaviors. Hum. Relat. 60, 1155–1179. doi: 10.1177/0018726707081658

[ref71] Thompson M. J. Carlson D. S. Kacmar K. M. Vogel R. M. (2019). The cost of being ignored: emotional exhaustion in the work and family domains. J. Appl. Psychol. 105, 186–195. doi: 10.1037/apl0000433, PMID: 31282700

[ref72] Thyer B. A. Myers L. L. (1998). Social learning theory. J. Hum. Behav. Soc. Environ. 1, 33–52. doi: 10.1300/J137v01n01_03

[ref73] Tiwari M. Tiwari T. Mishra L. Sundararaj V. Rajesh M. R. (2021). Corporate social responsibility and supply chain: a study for evaluating corporate hypocrisy with special focus on stakeholders. Int. J. Finan. Econ. 1–13. doi: 10.1002/ijfe.2483 [Epub ahead of print].

[ref74] Tokoro N. (2007). Stakeholders and corporate social responsibility (CSR): a new perspective on the structure of relationships. Asian Bus. Manag. 6, 143–162. doi: 10.1057/palgrave.abm.9200218

[ref75] Villalba-Ríos P. Barroso-Castro C. Vecino-Gravel J. D. (2022). The influence of CEO profile on corporate social responsibility companies. A qualitative comparative analysis. Corp. Soc. Respon. Environ. Manag. 29, 356–366. doi: 10.1002/csr.2205

[ref76] Wagner T. Lutz R. J. Weitz B. A. (2009). Corporate hypocrisy: overcoming the threat of inconsistent corporate social responsibility perceptions. J. Mark. 73, 77–91. doi: 10.1509/jmkg.73.6.77

[ref77] Wu T. J. Gao J. Y. Wang L. Y. Yuan K. S. (2020a). Exploring links between polychronicity and job performance from the person–environment fit perspective—The mediating role of well-being. Int. J. Environ. Res. Public Health 17:3711. doi: 10.3390/ijerph17103711, PMID: 32466109 PMC7277635

[ref78] Wu T. J. Wang L. Y. Gao J. Y. Wei A. P. (2020b). Social support and well-being of Chinese special education teachers – an emotional labor perspective. Int. J. Environ. Res. Public Health 17:6884. doi: 10.3390/ijerph17186884, PMID: 32967136 PMC7558049

[ref79] Wu Y. J. Wu T. (2019). Innovative work behaviors, employee engagement, and surface acting: a delineation of supervisor-employee emotional contagion effects. Manag. Decis. 57, 3200–3216. doi: 10.1108/MD-02-2018-0196

[ref80] Yang J. Diefendorff J. M. (2010). The relations of daily counterproductive workplace behavior with emotions, situational antecedents, and personality moderators: a diary study in Hong Kong. Pers. Psychol. 62, 259–295. doi: 10.1111/j.1744-6570.2009.01138.x

[ref81] Žukauskas P. Vveinhardt J. Andriukaitienė R. (2018). Corporate Social Responsibility as the Organization’s Commitment against Stakeholders. London: IntechOpen.

